# Adaptive Residual Weighted *K*-Nearest Neighbor Fingerprint Positioning Algorithm Based on Visible Light Communication

**DOI:** 10.3390/s20164432

**Published:** 2020-08-08

**Authors:** Shiwu Xu, Chih-Cheng Chen, Yi Wu, Xufang Wang, Fen Wei

**Affiliations:** 1 Key Laboratory of OptoElectronic Science and Technology for Medicine of Ministry of Education, College of Photonic and Electronic Engineering, Fujian Normal University, Fuzhou 350007, China; shiwuxu0501@163.com (S.X.); fzwxf@fjnu.edu.cn (X.W.); fafu.weifen@gmail.com (F.W.); 2 Concord University College, Fujian Normal University, Fuzhou 350117, China; 3 Department of Aeronautical Engineering, Chaoyang University of Technology, Taichung 413310, Taiwan; ccc@gm.cyut.edu.tw; 4 Information and Engineering College, Jimei University, Fujian 361021, China

**Keywords:** visible light communication, indoor positioning system, fingerprint positioning, weighted *K*-nearest neighbor, distance metric

## Abstract

The weighted *K*-nearest neighbor (WKNN) algorithm is a commonly used fingerprint positioning, the difficulty of which lies in how to optimize the value of *K* to obtain the minimum positioning error. In this paper, we propose an adaptive residual weighted *K*-nearest neighbor (ARWKNN) fingerprint positioning algorithm based on visible light communication. Firstly, the target matches the fingerprints according to the received signal strength indication (RSSI) vector. Secondly, *K* is a dynamic value according to the matched RSSI residual. Simulation results show the ARWKNN algorithm presents a reduced average positioning error when compared with random forest (81.82%), extreme learning machine (83.93%), artificial neural network (86.06%), grid-independent least square (60.15%), self-adaptive WKNN (43.84%), WKNN (47.81%), and KNN (73.36%). These results were obtained when the signal-to-noise ratio was set to 20 dB, and Manhattan distance was used in a two-dimensional (2-D) space. The ARWKNN algorithm based on Clark distance and minimum maximum distance metrics produces the minimum average positioning error in 2-D and 3-D, respectively. Compared with self-adaptive WKNN (SAWKNN), WKNN and KNN algorithms, the ARWKNN algorithm achieves a significant reduction in the average positioning error while maintaining similar algorithm complexity.

## 1. Introduction

Positioning systems can be divided into outdoor positioning system (OPS) and indoor positioning system (IPS). The OPS usually uses global positioning system (GPS) to obtain the coordinates of the target. Since the GPS signal is not able to penetrate the wall and other obstacles, GPS cannot be applied in the indoor positioning scene [1]. As a supplement to OPS, IPS has attracted increasing attention among researchers. At present, there are two main research areas on IPS. One is based on radio frequency communication technology, such as radio frequency identification (RFID) [2], wireless sensor network (WSN) [3], ultra-wideband (UWB) [4], wireless fidelity (WiFi) [5], Bluetooth [6], etc. The other is based on visible light communication (VLC) [7]. IPS can be divided into range-based IPS and range-free IPS. The methods of range-based include time of arrival (TOA), angle of arrival (AOA), and received signal strength indication (RSSI), etc. [8,9]. The range-free IPS usually uses fingerprint matching to achieve positioning [10]. Compared with radio frequency communication technology, using a light-emitting diode (LED) to achieve indoor positioning has the following advantages: (1) LED communication uses the visible light spectrum, which can be applied to some areas where electromagnetic radiation is prohibited, such as operating rooms and gas stations; (2) generally, LED is uniformly distributed on the ceiling, there is mainly line-of-sight (LoS) communication between transceivers and receivers; (3) existing LED lighting devices can be used directly, and the receiver can use integrated photodiode (PD) devices [11,12]; (4) the signal-to-noise ratio (SNR) is usually very high due to lighting requirements.

Typical fingerprint-based localization algorithms usually use machine-learning algorithms [13], for example, random forest (RF) [14], *K*-nearest neighbor (KNN) [15], extreme learning machine (ELM) [16], artificial neural network (ANN) [17], etc. In [11], for indoor positioning based on VLC, three classical machine-learning algorithms, RF, ELM and KNN are adopted to train multiple classifiers based on received signal strength indication (RSSI) fingerprints, and a grid-independent least square (GI-LS) algorithm was proposed to combine the outputs of these classifiers. Experimental results show that compared with RF, KNN and ELM algorithms, the positioning error based on the GI-LS algorithm is lower. In machine learning algorithms, *K*-nearest neighbor (KNN) is one of the most widely used fingerprint positioning. The KNN fingerprint positioning algorithm [15] works in two stages. The first one is run offline, and it consists of generating a set of fingerprint points in the application area. In the second step, the target measures an RSSI vector of *M* LEDs, which is then matched with the *K* nearest fingerprints obtained previously offline. When *K* fingerprints have different weights, this method is called the weighted *K*-nearest neighbor (WKNN) fingerprint positioning algorithm. The WKNN localization algorithm is based on the shortest physical distance between fingerprints and the target position [12,18,19], which usually adopts two ranging methods: Euclidean distance [18] and Manhattan distance [19]. In Hu et al. [19], for indoor positioning based on WiFi, a self-adaptive WKNN (SAWKNN) algorithm with a dynamic *K* was proposed. Experimental results show that the positioning error based on the SAWKNN algorithm is lower than that of the WKNN algorithm. In most cases, *M* LEDs are laid out on the ceiling of the same horizontal plane. The traditional trilateration method and least linear multiplication method can only solve the two-dimensional (2-D) coordinates of targets [20], and the height of the target from the floor needs to be known in advance, which is not feasible in many applications. A Newton–Raphson method was proposed in Şahin et al. [21] and Mathias et al. [22] to estimate the PD location. For a non-convex optimization problem of 3-D positioning, it is easy for the least linear multiplication method and Newton–Raphson method to fall into the local optimal solution, resulting in large positioning error. Particle swarm optimization [23] and differential evolution algorithm [24] are adopted to perform 3-D visible light positioning, which will increase the complexity of the algorithm. In Van et al. [25], compared with trilateration method in the case of ambient light interference and without ambient light interference, simulation results show that the positioning accuracy of the WKNN algorithm is improved by 36% and 50%, respectively. In Alam et al. [12], experiment results show that the average positioning error of the fingerprints established by Lambertian regeneration model is close to that of the actual RSSI measurement fingerprints, which are 2.7 cm and 2.2 cm, respectively. Therefore, the WKNN positioning algorithm based on VLC does not need a large number of human resources to acquire the fingerprints. In Gligorić et al. [26], a visible light localization algorithm based on compressed sensing (CS) was proposed. The orthogonal matching pursuit (OMP) reconstruction algorithm [27] is used to determine the overlapping region, and the KNN algorithm is used to determine the coordinates of the target. In Zhang et al. [28], an visible light inversion positioning system based on CS and a 4-sparse 2-D fingerprint matching algorithm was proposed. When CS is used to realize fingerprint positioning, the measurement matrix needs to satisfy the restricted isometry property (RIP) attribute, and the orthogonal decomposition of the measurement matrix is needed [29,30], which will increase the complexity of the algorithm. The fingerprint positioning algorithm based on CS must satisfy O (*K* log (*N*)), where this is the value of the number of measurements *M* (i.e., the number of LEDs) [29]. When the neighboring fingerprints *K* and the number of fingerprints *N* become larger, a high-density LED layout is required to satisfy the compression sensing reconstruction condition. However, an excessively dense LED layout not only wastes resources but also increases interference between LEDs. Although the WKNN algorithm is a commonly used fingerprint positioning, the difficulty lies in how to optimize the value of *K* to obtain the minimum positioning error. Compared with the traditional WKNN positioning algorithm, this paper makes the following contributions:

1. A novel adaptive residual weighted *K*-nearest neighbor (ARWKNN) fingerprint positioning algorithm is proposed, and *K* is a dynamic value according to the matched RSSI residual. As far as the authors know, most fingerprint positioning algorithms based on VLC only consider a fixed neighbor value, e.g., [12,25,28].
2. The impact of modulation bandwidth, transmit power, the signal-to-noise ratio, the maximum number of neighboring fingerprints, the sampling interval, the number of LEDs, the sampling ratio, and distance metric on positioning errors are analyzed in detail. The distribution of optimal *K* and the complexity of the algorithm are also analyzed in detail. The results can provide a useful reference for the design of the actual VLP system.
3. Simulation results show that the ARWKNN algorithm based on Clark distance (CLD) and minimum maximum distance (MMD) metrics produces the smallest average positioning error in 2-D and 3-D, respectively, as far as the authors know, this is the first work to report the impact of CLD and MMD metrics on the positioning error of the fingerprint positioning algorithm.
4. Simulation results show that when the SNR is 20 dB, in 2-D, compared with the fingerprint positioning algorithm based on RF [14], ELM [16], ANN [17], GI-LS [11], SAWKNN [19], WKNN [12] or KNN [15,25] algorithms, the average positioning error of the ARWKNN algorithm based on Manhattan distance can be reduced by 81.82%, 83.93%, 86.06%, 60.15%, 43.84%, 47.81%, and 73.36%, respectively. Compared with SAWKNN, WKNN and KNN algorithms, the ARWKNN algorithm can significantly reduce the average positioning error while maintaining similar algorithm complexity.

The rest of this paper is organized as follows: the ARWKNN algorithm is proposed in Section 2. Simulation results are shown and discussed in Section 3. Finally, Section 4 concludes this paper.

Notation: Matrices and vectors are in boldface. The field of real numbers is denoted by ℝ. ‖.‖_2_ is the 2 norm of the vector. |·| is the absolute value, and ⌈ ⌉ denotes the rounding up operator. The transpose operation is denoted by [.]^T^.

## 2. Design of the Adaptive Residual Weighted *K*-Nearest Neighbor (ARWKNN) Algorithm

### 2.1. System Model

**Figure 1:**
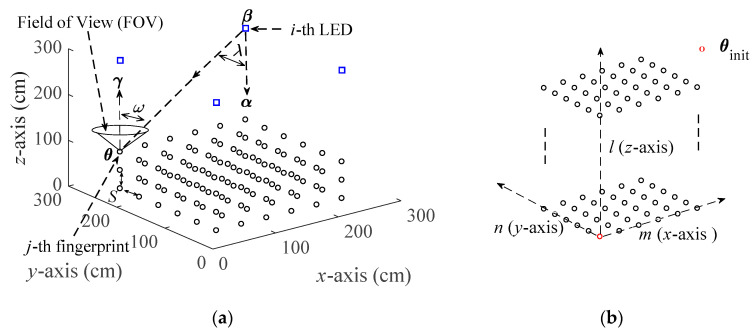
Fingerprint positioning based on visible light communication (VLC): (**a**) The positioning model; (**b**) The collection directions of fingeprints in *x*-axis, *y*-axis and *z*-axis.

The positioning model is shown in Figure 1a. If there is *M*_total_ LEDs in the room, the target checks and selects *M* LEDs that has the highest RSSI for positioning. For simplicity, we assume that the target appears in a 3-D space with *M* LEDs. The coordinates of *M* LEDs are **β***_i_* = [*x*_LED-*i*_, *y*_LED-*i*_, *z*_LED-*i*_]^T^, for *i* = 1, 2, …, *M*. It is assumed that *M* LEDs are evenly distributed on the same horizontal plane, i.e., *z*_LED-*i*_ = *z*_LED_, *z*_LED_ is the height from the floor to the LED. $\alpha_{i}\in {\mathbb{R}}^{3\times 1}$ represents the angle of the *i*th LED. **θ***_j_*$\in {\mathbb{R}}^{3\times 1}$ and **γ***_j_*$\in {\mathbb{R}}^{3\times 1}$ represent the coordinate and angle of the *j*th fingerprint point, respectively, for *j* = 1, 2, …, *N*, *N* represents the number of fingerprint points. Suppose the target moves in an interval from *h*_L_ to *h*_H_ at the z-axis, *h*_L_ and *h*_H_ are the minimum and maximum vertical distance from the floor to the target, respectively.

We use *S* to denote the spacing of the fingerprints, as shown in Figure 1a. *m*, *n* and *l* are used to represent the collection directions of fingerprints in *x*-axis, *y*-axis and *z*-axis, respectively, the meanings of *m*, *n*, and *l* are shown in Table 1. To make it easier to understand, an example is given, as shown in Figure 1b. In Figure 1b, columns are arranged from left to right (in the positive direction of the *x*-axis), rows are arranged from bottom to top (in the positive direction of the *y*-axis), and dimensions are arranged from low to high (in the positive direction of the *z*-axis). The starting point of fingerprint collection is **θ**_init_ = [*x*_init_, *y*_init_, *z*_init_]^T^, *x*_init_, *y*_init_, and *z*_init_ are given by:

(1) $$\left\{ \begin{array}{l} x_{\mathrm{init}}=\min\left(x_{\mathrm{LED}-i}\right)\\ y_{\mathrm{init}}=\min\left(y_{\mathrm{LED}-i}\right)\\ z_{\mathrm{init}}=h_{L} \end{array}\right.$$

Then, in the positioning space, the coordinates corresponding to the fingerprint points in the *l* dimension, i.e., the *m* column and the *n* row are

(2) $$\left\{ \begin{array}{l} x_{\mathrm{fin}-m}=x_{\mathrm{init}}+S\left(m-1\right),\;m=1,\;2,\;\ldots \;,\;\left\lceil \frac{L_{1}}{S}+1\right\rceil \\ y_{\mathrm{fin}-n}=y_{\mathrm{init}}+S\left(n-1\right),\;\;\;\;n=1,\;2,\;\ldots \;,\;\left\lceil \frac{L_{2}}{S}+1\right\rceil \\ z_{\mathrm{fin}-l}=z_{\mathrm{init}}+S\left(l-1\right),\;\;\;\;\;\;l=1,\;2,\;\ldots \;,\;\left\lceil \frac{L_{3}}{S}+1\right\rceil \end{array}\right.$$

where *L*_1_ = max (*x*_LED-*i*_) − min (*x*_LED-*i*_), *L*_2_ = max (*y*_LED-*i*_) − min (*y*_LED-*i*_) and *L*_3_ = *h*_H_ − *h*_L_. Then the distance *d_l_*_,*m*,*n*−*i*_ between each fingerprint point and the *i*th LED can be obtained as:

(3) $$d_{l,m,n-i}=\sqrt{{\left(x_{\mathrm{fin}-m}-x_{\mathrm{LED}-i}\right)}^{2}+{\left(y_{\mathrm{fin}-n}-y_{\mathrm{LED}-i}\right)}^{2}+{\left(z_{\mathrm{fin}-l}-z_{\mathrm{LED}-i}\right)}^{2}}$$

### 2.2. Fingerprint Matrix Construction

We use $\Phi \in {\mathbb{R}}^{M\times N}$ to denote the measurement matrix of the fingerprints, which is given by:

(4) $$\Phi =\left(\begin{array}{l} \phi_{1,1,1-1}\;\phi_{1,1,2-1}\;\cdots \;\phi_{l,m,n-1}\\ \phi_{1,1,1-2}\;\phi_{1,1,2-2}\;\cdots \;\phi_{l,m,n-2}\\ \;\;\;\;\;\;\;\;\vdots \;\;\;\;\;\;\;\;\vdots \;\;\;\;\;\;\;\;\ddots \;\;\;\;\;\;\;\;\vdots \\ \phi_{1,1,1-M}\;\phi_{1,1,2-M}\;\cdots \;\phi_{l,m,n-M} \end{array}\right)$$

where *N* is given by:

(5) $$N=\left\lceil \frac{L_{1}}{S}+1\right\rceil \left\lceil \frac{L_{2}}{S}+1\right\rceil \left\lceil \frac{L_{3}}{S}+1\right\rceil$$

And $\phi_{l,m,n-i}$ represents the RSSI, which is given by:

(6) $$\phi_{l,m,n-i}=10\log_{10}\left(P_{l,m,n-i}\right)$$

where *P_l,m,n_*_−*i*_ represents the optical power value from the *i*th LED received by the fingerprint point in the *l* dimension, *m* column and *n* row within the positioning area.

### 2.3. Measurement Vector

Suppose the coordinates of targets in 3-D are **Ψ***_k_* = [*x*_target-*k*_, *y*_target-*k*_, *z*_target-*k*_]^T^, for *k* = 1, 2, …, *C*, and *C* represents the number of targets. Thus, the receiving signal intensity vector **Y***_k_* of *M* LEDs collected by the *k*th target is given by:

(7) $$Y_{k}={\left[Y_{k,1},Y_{k,2},\;\ldots \;,\;Y_{k,M}\right]}^{T}$$

where *Y_k_*_,*i*_ is given by

(8) $$Y_{k,i}=10\log_{10}\left(P_{k,i}\right)$$

where *P_k_*_,*i*_ represents the optical power value of the *i*th LED received by the *k*th target.

### 2.4. Measurement Model

In this paper, the measurement matrix **Φ** and measurement vector **Y**_k_ are generated by the Lambertian radiation model. Because the LED is distributed on the ceiling, there is mainly LoS communication between the fingerprint point and the LED. Without loss of generality, this paper only considers the Lambertian radiation model of the LoS, which are widely adopted in papers such as [12,28,30,31,32], the received light power value of the fingerprint point is:

(9) PRe=PTrAPD(b+1)Tsg2πd2(cos(λi))bcos(ωi)

where *P*_Re_ represents the received light power value; *P*_Tr_ represents the transmit power of the LED; *d* is the distance between the transmitter and the receiver; *T_s_* and *g* are the optical filter gain and optical concentrator gain, respectively; *b* is the Lambertian order; *λ*_1/2_ is the half-power angles of the LED; *A*_PD_ is the effective area of the PD detection; The field of view (FOV) of PD is defined as *ω*_FOV_, and 0 < *ω_i_* < *ω*_FOV_. *λ_i_* and *ω_i_* are the radiation and incident angles, i.e., the transmitter’s normal and receiver’s normal, respectively, as shown in Figure 1a.

### 2.5. Channel Access Method

**Figure 2:**
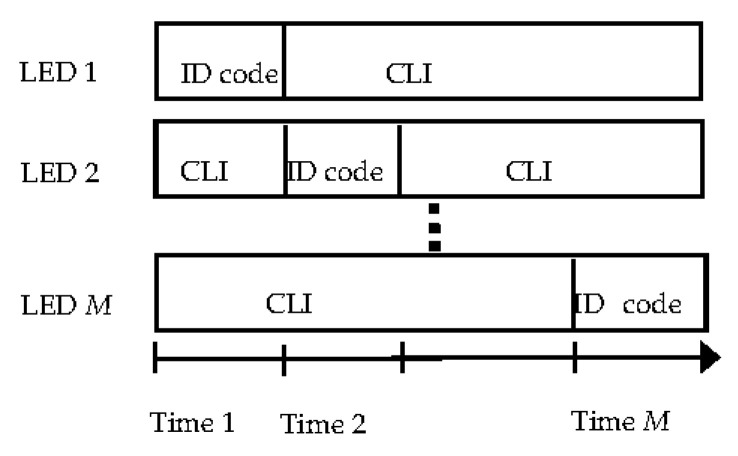
Frame structure of the positioning system for one period.

As LEDs transmit a unique identification (ID) code independently, however, signals sent from different LEDs will interfere with each other at the receiver. In order to receive the power from different LEDs, we also use time division multiplexing to achieve this goal [20,31,32], and in a real scenario, we can also use different modulation frequencies, such as Guo et al. [11] and Alam et al. [12]. *M* LEDs have synchronous frames [20,31], and different LEDs use different time slots to transmit signals within each frame cycle, when one LED transmits the ID code, other LEDs emit a constant light intensity (CLI) for illumination purposes only. The frame structure is shown in Figure 2. After photoelectric conversion, a high-pass filter can be used to filter out the power from other LEDs [20].

### 2.6. Setting of K

**Figure 3:**
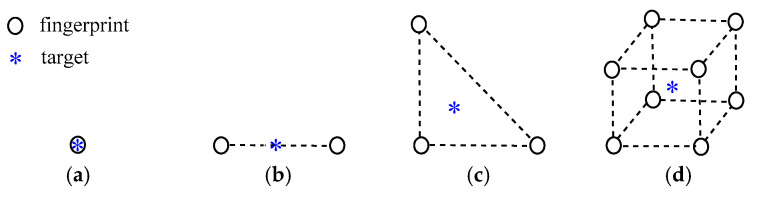
The relationship between target and fingerprints: (**a**) *K* = 1; (**b**) *K* = 2; (**c**) *K* = 3; (**d**) *K* = 8.

According to the principle of fingerprint positioning, the purpose of positioning is to find *K* fingerprint points that are close to the target. When in a different experimental environment, *K* generally takes different values, such as in Xue et al. [15], the optimal positioning accuracy is obtained when *K* = 5; in Alam et al. [12] and Zhang et al. [28], the optimal positioning accuracy is obtained when *K* = 4; in Van et al. [25], the optimal positioning accuracy is obtained when *K* = 3. One thing they all have in common is that *K* is a fixed value. In this paper, *N* fingerprint points are evenly distributed in the 2-D or 3-D space. In a specific time, there are *K* fingerprint points close to the same target, which is called the KNN fingerprint positioning algorithm. For example, when the target exactly matches the fingerprint point, as shown in Figure 3a, obviously, the optimal positioning accuracy is obtained when *K* = 1. When the target falls on a straight line formed by two fingerprint points, as shown in Figure 3b, i.e., *K* = 2. When the target is in a triangular area composed of three fingerprint points, as shown in Figure 3c, i.e., *K* = 3. If the 3-D fingerprints map is adopted, and the target is obviously located in a minimum cube composed of 8 fingerprint points with a high probability, i.e., *K* = 8, as shown in Figure 3d.

**Figure 4:**
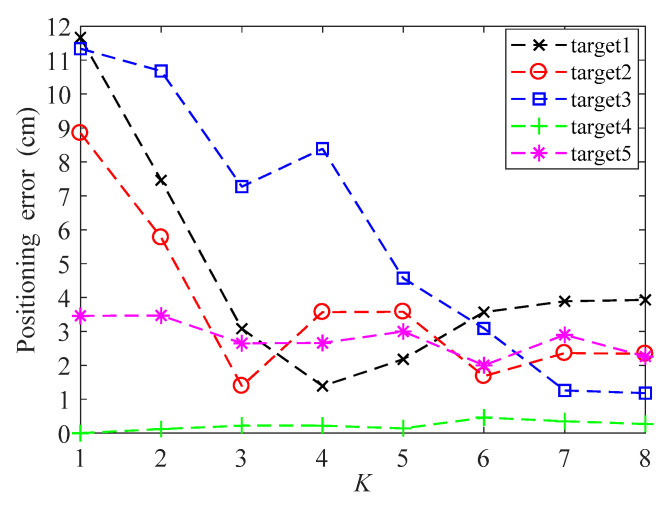
Positioning errors of 5 targets using weighted *K*-nearest neighbor (WKNN) algorithm.

Figure 4 is the positioning error of five targets at different 3-D positions using WKNN algorithm, for *K* increases from 1 to 8. When *K* = 4, the positioning error of target 1 is minimal. When *K* = 3, the positioning error of target 2 is minimal. When *K* = 8, the positioning error of target 3 is minimal. When *K* = 1, the positioning error of target 4 is minimal. When *K* = 6, the positioning error of target 5 is minimal. It can also be seen from Figure 4 that the positioning error varies with the *K* value fluctuation, and there is no monotonous increasing or decreasing relationship. In a 2-D visible light localization, the average positioning error based on the WKNN algorithm can be minimized when *K* = 3 or *K* = 4, e.g., [12,25,28]. In the 3-D visible light localization, the average positioning error based on the WKNN algorithm can be minimized when *K* = 8, which will be discussed in Section 3 . The minimum mean positioning error does not mean that the positioning error of each target is the smallest, so the dynamic *K* value can effectively reduce the positioning error of different targets. To address this issue, this paper proposes an adaptive residual weighted *K*-nearest neighbor fingerprint positioning algorithm, which is called ARWKNN fingerprint positioning algorithm.

### 2.7. ARWKNN Algorithm

The WKNN fingerprint positioning algorithm is based on the shortest RSSI physical distance between the fingerprint and the target position. The positioning error for the WKNN algorithm is affected by the weight of the fingerprint point and this weight is affected by the *K* value. If the optimal *K* value can be obtained, the positioning error can be reduced, so a novel ARWKNN algorithm is proposed in this paper. The pseudo-code of the ARWKNN algorithm is shown in Algorithm 1. In Algorithm 1, if we only consider Steps 1, 2 and 5, then it is the WKNN algorithm, and in Step 5, if the location of the target is estimated by averaging the coordinates of *K* fingerprints, then it is the KNN algorithm. By contrast with the KNN and WKNN algorithms, the ARWKNN algorithm also performs Step 3 and 4 in Algorithm 1. Because there is no prior information about the location of the target, that is, the value of **Ψ***_k_* is unknown, but we known the fingerprint matrix **Φ** and the target RSSI measurement vector **Y***_k_*, we can adaptively select the *K* value by matching the residual between the measured and calculated RSSI values. Therefore, the purpose of Steps 3 and 4 in algorithm 1 is to obtain the optimal *K* value, i.e., the *K* value corresponding to the smallest RSSI matching residual. In Algorithm 1, because the maximum number of neighboring fingerprint points *K*_max_ is much smaller than the total number of fingerprint points *N*, the ARWKNN algorithm has a large reduction in the average positioning error while maintaining similar algorithm complexity, which will be discussed in Section 3.4.
**Algorithm 1.** ARWKNN algorithm**Input:** the maximum number of nearest neighbor fingerprints *K*_max_, fingerprint matrix **Φ**, and the *k*th target measurement vector **Y***_k_*. **Output:** The coordinates of the *k*th target, i.e., **Ψ***_k_*.**Step 1:** Calculate the distance from the *k*th target to *N* fingerprint points.$dis_{l,m,n-k}={\left(\sum_{i=1}^{M}{\left|\phi_{l,m,n-i}-Y_{k,i}\right|}^{r}\right)}^{1/r}$
where *r* = 1 represents the Manhattan distance, *r* = 2 represents the Euclidean distance. **Step 2:** Sort the distance values in ascending order, i.e.,
[**X**, **I**] = sort (**dis**).
where **dis** = [*dis*_1,1,1−*k*_, *dis*_1,1,2−*k*_, …, *dis_l_*_,*m*,*n*−*k*_]^T^$\in {\mathbb{R}}^{N\times 1}$, $X\in {\mathbb{R}}^{N\times 1}$ represents the distance vector after sorting, and $I\in {\mathbb{R}}^{N\times 1}$ represents the corresponding index set. **Step 3:** Calculate the matched RSSI residuals. *K* = 1, **while**
$K\leq K_{\max}$
**do**  **for**
*ii* = 1: *K*  A(:,ii)=Φ(:,I(ii));  **end for** where $A\in {\mathbb{R}}^{M\times K}$ represents finding the *K* column values corresponding to the fingerprint matrix **Φ** according to the index set **I**. Calculate the *k*th target RSSI vector via *K* nearest neighbor fingerprints,$${\tilde{Y}}_{k}=AB,$$
where **B** = [*B*_1_, *B*_2_, …, *B_K_*]^T^ ∈${\mathbb{R}}^{K\times 1}$ and $B_{t}=\frac{1}{X\left(t\right)}/\sum_{tt=1}^{K}\frac{1}{X(tt)}$, for *t* = 1, 2, …, *K*, Calculate the matched RSSI residual between the measured and calculated RSSI values,$$E_{\mathrm{residual}}=Y_{k}-{\tilde{Y}}_{k},$$
and calculate the sum of the absolute values of the residuals,$E_{\mathrm{sum}}\left(K\right)=\sum_{i=1}^{M}\left|E_{\mathrm{residual}}\left(i\right)\right|,$
*K* = *K* + 1. **end while** **Step 4:** Output the *K* value, i.e.,$K=\arg\min\left(E_{\mathrm{sum}}\right);\;s.t.\;1\leq K\leq K_{\max}$
**Step 5:** Calculate the coordinates of the *k*th target,$\Psi_{k}=\frac{\sum_{t=1}^{K}\frac{1}{X\left(t\right)}\theta_{I\left(t\right)}}{\sum_{t=1}^{K}\frac{1}{X\left(t\right)}}$
where **θ_I_**_(*t*)_ represents the coordinates of the corresponding fingerprint point found according to the index set **I**.

## 3. Simulation Analysis

In this Section, the ARWKNN algorithm is compared with RF [14], ELM [16], ANN [17], GI-LS [11], SAWKNN [19], WKNN [12] or KNN [15,25] algorithms. The basic principle of the fingerprint positioning algorithm based on RF, ELM, ANN, and GI-LS machine learning is as follows [11,13]: Firstly, the positioning area is divided into several equal grid points according to the sampling interval *S*, RSSI measurements are obtained by placing the receiver at different grid points, and each grid point represents a category. Secondly, machine-learning algorithms are used to train the category to which each grid point belongs. Thirdly, the RSSI measurements obtained in the online phase are compared with the derived model to predict the location of the target.

### 3.1. Error Definition

Suppose the actual coordinates of targets are ${\tilde{\Psi }}_{k}\in {\mathbb{R}}^{3\times 1}$, then the positioning error *E_k_* is defined as:

(10) $$E_{k}={\left\|\Psi_{k}-{\tilde{\Psi }}_{k}\right\|}_{2}$$

and the average positioning error *E*_APE_ is defined as:

(11) $$E_{\mathrm{APE}}=\frac{1}{C}\sum_{k=1}^{C}E_{k}$$

### 3.2. Noise Model of Visible Light Communication (VLC)

In indoor VLC, the noise *σ*_noise_ includes shot noise *σ*_shot_ and thermal noise *σ*_thermal_ [33], which are given by:

(12) $$\sigma_{\mathrm{noise}}^{2}=\sigma_{\mathrm{shot}}^{2}+\sigma_{\mathrm{thermal}}^{2}$$

(13) $$\sigma_{\mathrm{shot}}^{2}=2qR_{\mathrm{PD}}P_{r}B+2qI_{bg}I_{2}B$$

(14) $$\sigma_{\mathrm{thermal}}^{2}=\frac{8\pi k_{B}T_{K}}{G_{0}}\eta A_{PD}I_{2}B^{2}+\frac{16\pi^{2}k_{B}T_{K}\Gamma }{g_{m}}\eta^{2}A_{PD}^{2}I_{3}B^{3}$$

where *q* is elementary charge, *R*_PD_ is the responsivity of the PD, *B* is the equivalent noise bandwidth, *P*_r_ indicates the received power from *M* LEDs, *k_B_* is the Boltzmann’s constant, *T*_K_ is the absolute temperature, *G*_0_ is the open loop gain, *η* is the fixed capacitance of PD, *I*_bg_ is the background light current, Γ is the channel noise factor, *g_m_* is the field effect transistor (FET) transconductance, *I*_2_ and *I*_3_ are the noise bandwidth factors.

According to the noise model, the signal-to-noise ratio (SNR) is given by [32]

(15) $$\mathrm{SNR}\left(\mathrm{dB}\right)=10\log_{10}\frac{{\left(R_{\mathrm{PD}}\frac{P_{r}}{A_{\mathrm{PD}}}\right)}^{2}}{\sigma_{\mathrm{noise}}^{2}}$$

### 3.3. Simulation Parameters

Without loss of generality, we suppose **α***_i_* = [0, 0, −1]^T^ and **γ***_j_* = [0, 0, 1]^T^, i.e., cos(*λ_i_*) = cos(*ω_i_*) = *h_l_*_,*m*,*n*-*i*_/*d_l_*_,*m*,*n*-*i*_, *h_l_*_,*m*,*n*-*i*_ is the *z*-axis distance from the fingerprint point to the *i*th LED in the *l* dimension, the *m* column and the *n* row, which are widely adopted in papers such as [12,20,28]. The parameter setting of the Lambertian radiation model is as follows: *T*_s_ = *g* = 1, *λ*_1/2_ = π/3, *ω*_FOV_ = π/2, *A*_PD_ = 1 cm^2^, *b* = 1, which follow from a typical LED setup. *M* LEDs are evenly distributed in a 3-D space with an area of 200 cm × 200 cm × 300 cm, min (*x*_LED-*i*_) = min (*y*_LED-*i*_) = 0 cm, max (*x*_LED-*i*_) = max (*y*_LED-*i*_) = 200 cm, and *z*_LED_ = 300 cm. *C* = 200, i.e., 200 targets randomly appear in the 3-D or 2-D positioning area. In the KNN and WKNN algorithms, *K* is a fixed value, that is, *K* = *K*_max_. The parameter setting of the noise model is as follows [33]: *T*_K_ = 295 K, *G*_0_ = 10, *g_m_* = 30 mS, Γ = 1.5, *I*_2_ = 0.562, *I*_3_ = 0.0868, *R*_PD_ = 0.54 A/W, *η* = 112 pF/cm^2^, *I*_bg_ = 5100 *µ*A. Unless otherwise specified, *P*_Tr_ = 6 W, *M* = 4, *r* = 1 (i.e., Manhattan distance). In 3-D, *h*_L_ = 20 cm and *h*_H_ = 100 cm. In 2-D, *h*_L_ = *h*_H_ = 100 cm. The simulation tool is MATLAB R2017a.

**Figure 5:**
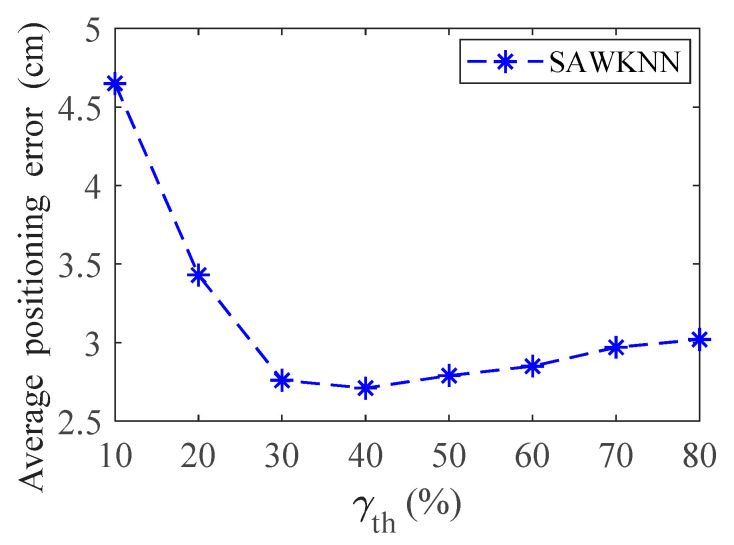
The impact of *γ*_th_ on the average positioning error.

For simplicity, unless otherwise specified, we only consider the 2-D case, and *S* = 20 cm. In order to obtain the optimal classification accuracy of ANN, ELM, and RF algorithms, and the optimal positioning accuracy of KNN, WKNN, and SAWKNN algorithms. The optimal parameters obtained through offline training and learning are as follows: In KNN, WKNN, ARWKNN and SAWKNN algorithms, *K*_max_ = 4. In the Section 3.4, we will also discuss the impact of different *K*_max_ values on the average positioning error. For the optimal number of hidden nodes and trees, the classification method is the same as that in Guo et al. [11], i.e., each grid point represents a category, and the cross-validation method is adopted based on experience adjustment. For the optimal number of hidden nodes, the cross-validation method has a range of 100 to 700 and a step size of 50. For the optimal number of trees, the cross-validation method has a range of 10 to 50 and a step size of 5. After comprehensive evaluation of the positioning accuracy and classification accuracy, the optimal number of hidden nodes and trees are selected to be 600 and 40, respectively. The impact of *γ*_th_ on the average positioning error is shown in Figure 5, it can be seen from the Figure 5 that minimum average positioning error is achieved when *γ*_th_ is within the range of [30%, 50%], so, the value of *γ*_th_ is selected to be 40%, which denotes the threshold of two RSSI difference values that can be considered similar [19].

### 3.4. Result Analysis

We only consider positioning in this paper, so *B* = 640 KHz will be able to label 3.4 × 10^38^ LEDs [34], which is far exceeds the actual needs. The SNR experimental results are shown in Table 2. If *B* = 640 KHz, typical SNR for indoor visible light communication ranges from 42.97 to 60.92 dB, and the average value reaches 52.45 dB. In addition to indoor positioning, LEDs can also provide high-speed data rate, If *B* = 100 MHz, the average SNR can also reach 28.86 dB.

**Figure 6:**
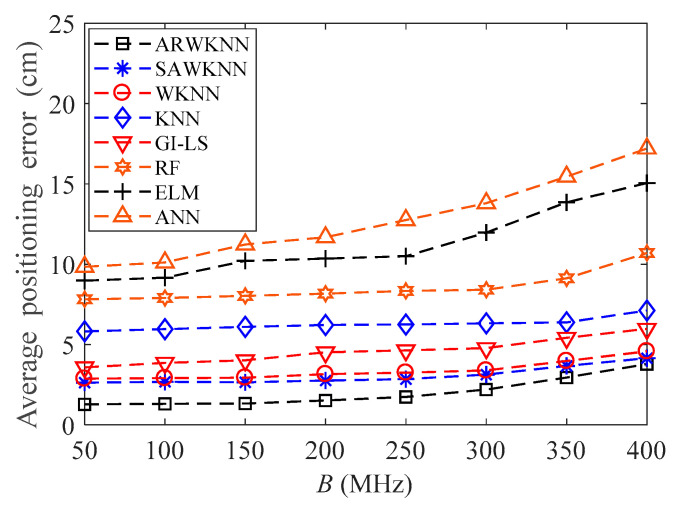
The impact of *B* on the average positioning error with *K*_max_ = 4.

When *P*_tr_ = 6 W, the average positioning errors of eight algorithms are analyzed when *B* is within 50 MHz to 400 MHz, the results are shown in Figure 6. As the value of modulation bandwidth increases, the average positioning errors of eight algorithms increase. The higher the modulation bandwidth, the lower the SNR and the higher the average positioning errors. As only positioning is considered in this paper, a very high modulation bandwidth is not necessary. With a high-modulation bandwidth, it may be more suitable to modulate the transmission signal of the LED by modified orthogonal frequency division multiplexing (OFDM) to achieve indoor positioning [22,35,36], but this is beyond the scope of this paper. It can also be seen from Figure 6 that when *B* is within 50 MHz to 400 MHz, the average positioning error based on the ARWKNN algorithm is the smallest.

**Figure 7:**
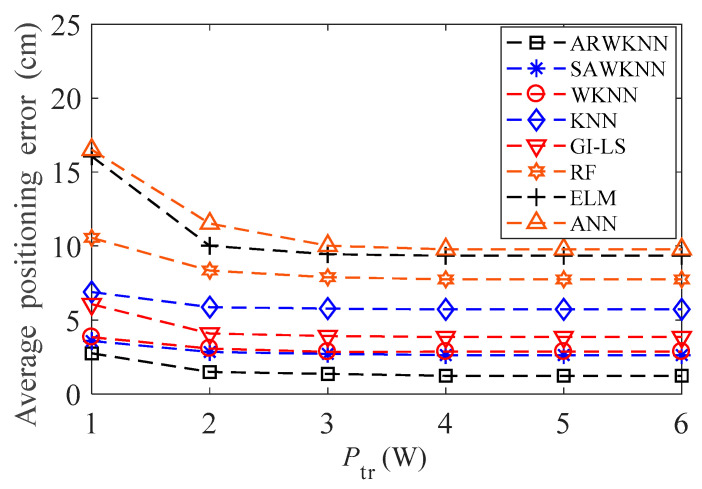
The impact of *P*_tr_ on the average positioning error with *K*_max_ = 4.

When *B* = 100 MHz, the average positioning errors of eight algorithms are analyzed when *P*_tr_ is within 1 W to 6 W, the results are shown in Figure 7. As the *P*_tr_ increases, the average positioning errors of eight algorithms decrease. When *P*_tr_ = 3 W, the average positioning errors of eight algorithms are close to convergence. The higher the transmitting power, the higher the SNR and the smaller the average positioning errors. It can also be seen from Figure 7 that when *P*_tr_ is within 1 W to 6 W, the average positioning error based on the ARWKNN algorithm is the smallest.

**Figure 8:**
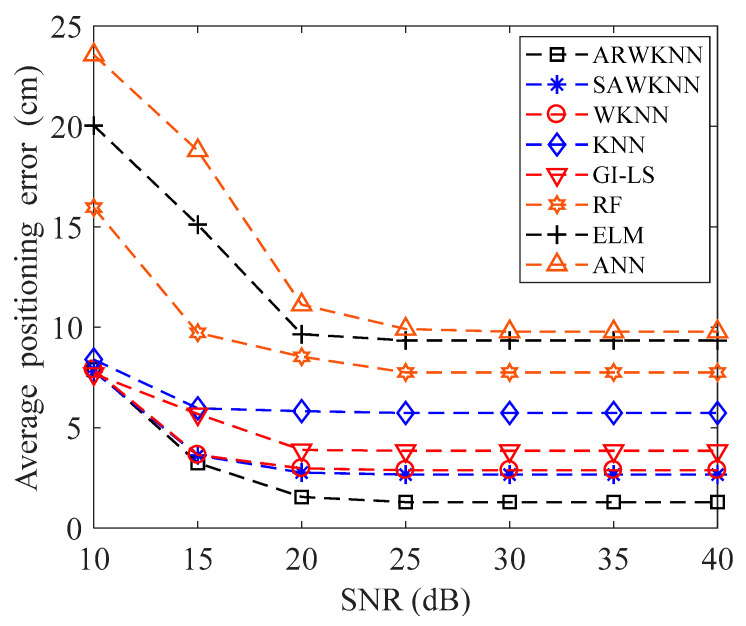
The impact of SNR on the average positioning error with *K*_max_ = 4.

The average positioning errors of eight algorithms under different SNR are compared, simulation results are shown in Figure 8. As shown in Figure 8, when SNR = 10 dB, the average positioning errors of eight algorithms are large due to severe noise interference. As the SNR increases, the average positioning errors of eight algorithms decrease. When SNR = 20 dB, the average positioning errors of eight algorithms are close to convergence. Since fingerprint positioning based on RF, ELM and ANN algorithms can only determine the category of the target, compared with WKNN algorithm, the positioning error is larger. When the SNR is higher than 15, the average positioning error based on the ARWKNN algorithm is the smallest. Due to lighting requirements and LoS communication, within the typical SNR range of indoor visible light communication, the average positioning error based on the ARWKNN algorithm is significantly lower than that of RF, ELM, ANN, GI-LS, SAWKNN, WKNN and KNN algorithms. The average positioning error based on the SAWKNN algorithm is lower than that of the WKNN algorithm. The GI-LS algorithm uses the complementary advantages of KNN, RF, and ELM classifiers to weight the estimation results, the average positioning error based on the GI-LS algorithm is lower then that of KNN, RF, ELM and ANN algorithms, but higher then WKNN, ARWKNN and SAWKNN algorithms.

When SNR = 20 dB, the average positioning errors based on ARWKNN, RF, ELM, ANN, GI-LS, SAWKNN, WKNN and KNN algorithms are shown in Table 3. It can be seen from Table 3 that compared with RF, ELM, ANN, GI-LS, SAWKNN, WKNN and KNN algorithms, the average positioning error based on the ARWKNN algorithm can be reduced by 81.82%, 83.93%, 86.06%, 60.15%, 43.84%, 47.81%, and 73.36%, respectively.

**Figure 9:**
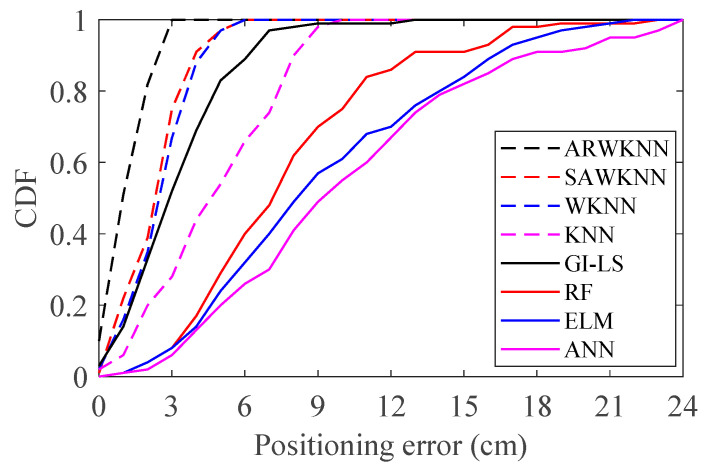
The cumulative distributions of positioning errors with *K*_max_ = 4.

When SNR = 20 dB, the simulation results of cumulative distribution function (CDF) are shown in Figure 9. It can be seen from Figure 9 that the CDF of positioning errors based on the ARWKNN algorithm is significantly better than that of the RF, ELM, ANN, GI-LS, SAWKNN, WKNN and KNN algorithms. The KNN algorithm is one of the simplest of all machine learning algorithms. Compared with the RF, ELM, ANN and GI-LS algorithms, fingerprint positioning based on the ARWKNN algorithm, not only has lower complexity, but also has lower positioning error. Fingerprint positioning is based on machine-learning algorithms, which require a large amount of data for training and learning. If there are not enough training data, the positioning error will be large, and a large amount of training data will increase the complexity of the algorithm. Compared with the SAWKNN, WKNN, and KNN algorithms, the ARWKNN algorithm can significantly reduce the average positioning error while maintaining similar algorithm complexity, which will be discussed in the section of algorithm complexity analysis.

**Figure 10:**
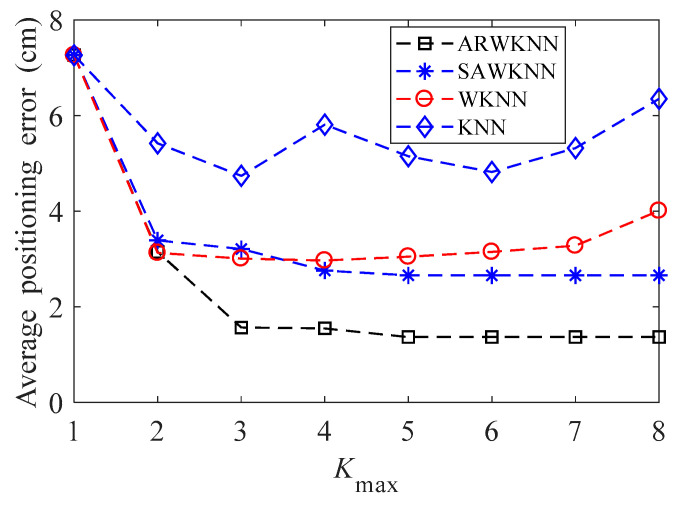
In 2-D, the impact of *K*_max_ on the average positioning error.

**Figure 11:**
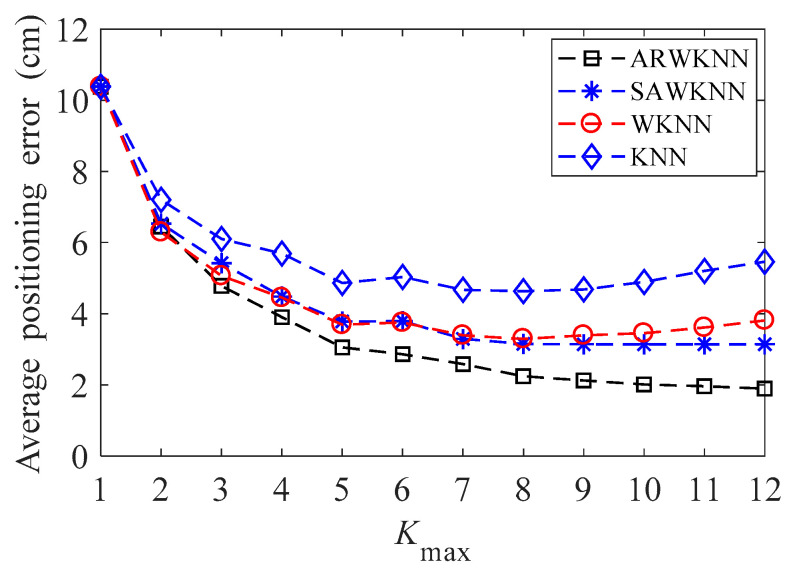
In 3-D, the impact of *K*_max_ on the average positioning error.

When SNR = 20 dB, the average positioning errors of ARWKNN, SAWKNN, WKNN, and KNN algorithms are analyzed, in WKNN and KNN algorithms, *K* is a fixed value, that is, *K* = *K*_max_. The simulation results of 2-D and 3-D are shown in Figure 10 and Figure 11, respectively. As can be seen from Figure 10, when *K*_max_ is within 1 to 8, similar to the experimental results in most papers, in 2-D, the optimal *K* based on the WKNN algorithm is 3 or 4, which exactly conforms with the fact that the target will be located in a minimum triangle or square composed of 3 or 4 fingerprint points with a high probability. It can also be analyzed from Figure 10 that when *K*_max_ is greater than 3, the average positioning error based on the ARWKNN algorithm is significantly lower than that of the KNN, WKNN, and SAWKNN algorithms. From Figure 11, It can be seen that as the *K*_max_ increases from 1 to 12, the average positioning error based on the ARWKNN algorithm decreases. When *K*_max_ = 8, the average positioning error is not significantly reduced if the value of *K*_max_ continues to increase. Therefore, a reasonable value of *K*_max_ is taken as 8. From Figure 11, we can also see that when *K*_max_ = 8, the average positioning error based on the KNN and WKNN algorithms is the smallest, which exactly conforms that the target will be located in a minimum cube composed of 8 fingerprint points with a high probability. It can also be analyzed from Figure 11 that when *K*_max_ is greater than 6, the average positioning error based on the ARWKNN algorithm is significantly lower than that of the KNN, WKNN, and SAWKNN algorithms, and the advantages of the ARWKNN algorithm are more obvious as *K*_max_ increases.

**Figure 12:**
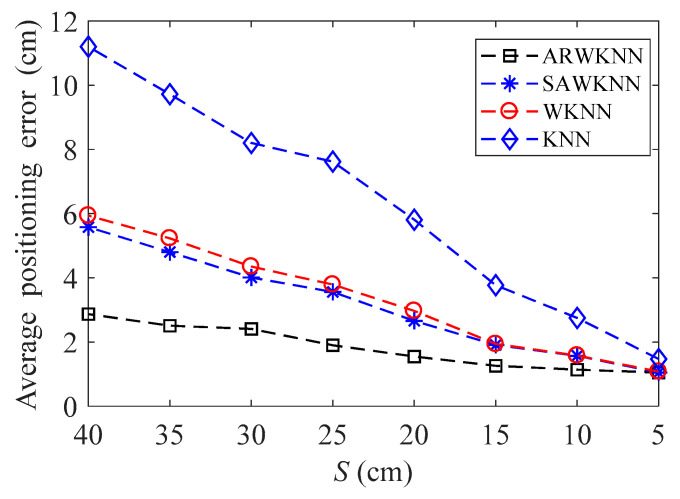
In 2-D, the impact of *S* on the average positioning error with *K*_max_ = 4.

**Figure 13:**
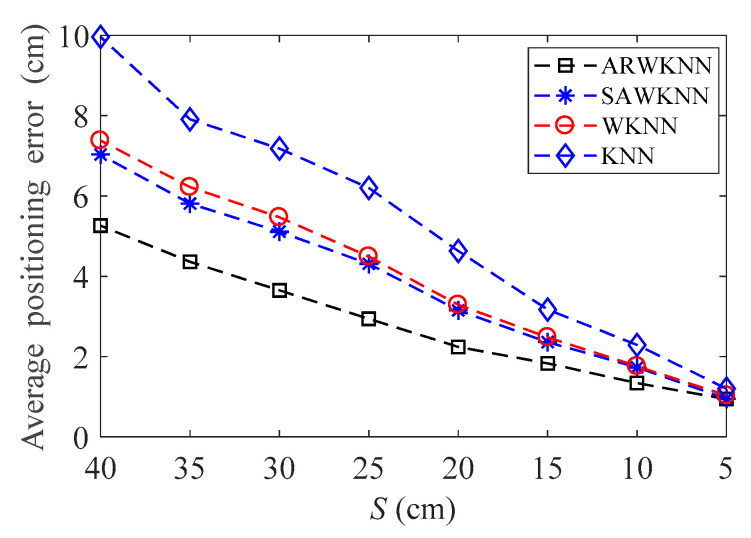
In 3-D, the impact of *S* on the average positioning error with *K*_max_ = 8.

When SNR = 20 dB, the average positioning errors of ARWKNN, SAWKNN, WKNN, and KNN algorithms are analyzed with the variation of the fingerprint sampling point spacing *S*, the results of 2-D and 3-D are shown in Figure 12 and Figure 13, respectively. It can be seen that as *S* decreases from 40 cm to 20 cm, whether in 2-D or 3-D, the average positioning error based on the ARWKNN algorithm is significantly lower than that of the KNN, WKNN, and SAWKNN algorithms, and the larger the *S*, the more obvious the advantage. As *S* decreases to 5 cm, the average positioning errors of four algorithms tend to be the same. The lower the value of *S*, the larger the number of fingerprint points *N* to be acquired, and the more complicated the algorithm becomes.

**Figure 14:**
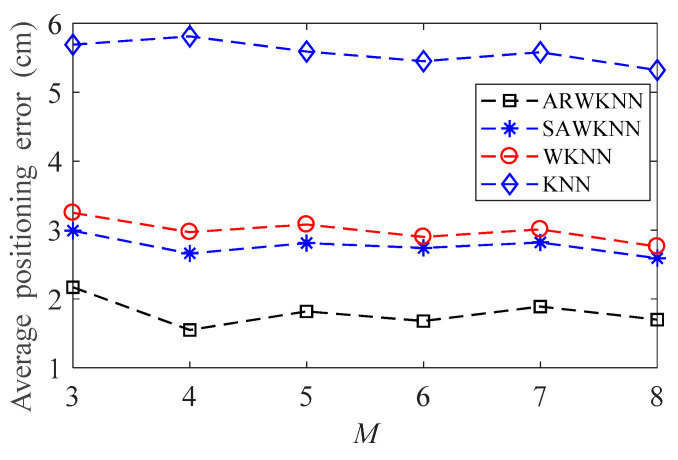
In 2-D, the impact of *M* on the average positioning error with *K*_max_ = 4.

**Figure 15:**
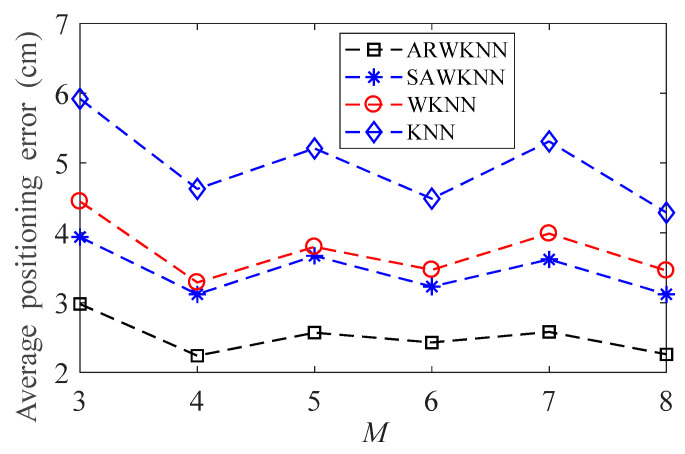
In 3-D, the impact of *M* on the average positioning error with *K*_max_ = 8.

When SNR = 20 dB, the average positioning errors of ARWKNN, SAWKNN, WKNN, and KNN algorithms are analyzed when *M* is within 3 to 8, the results of 2-D and 3-D are shown in Figure 14 and Figure 15, respectively. It can be seen that as *M* increases from 3 to 8, the average positioning errors based on the KNN, WKNN, SAWKNN and ARWKNN algorithms do not change much. Thus, only 4 LEDs are needed to achieve very low positioning error in this paper.

**Figure 16:**
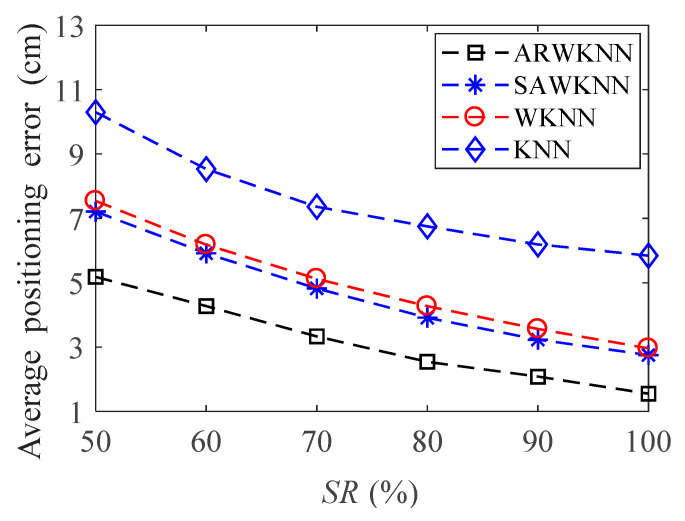
In 2-D, the impact of sampling ratio (*SR*) on the average positioning error with *K*_max_ = 4.

**Figure 17:**
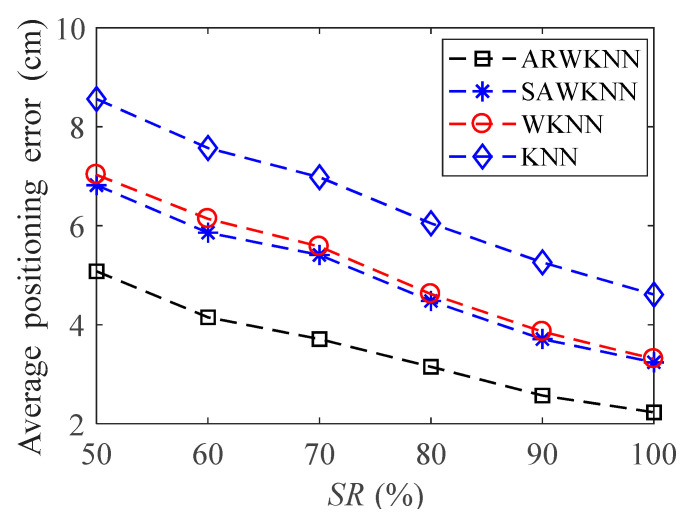
In 3-D, the impact of *SR* on the average positioning error with *K*_max_ = 8.

**Figure 18:**
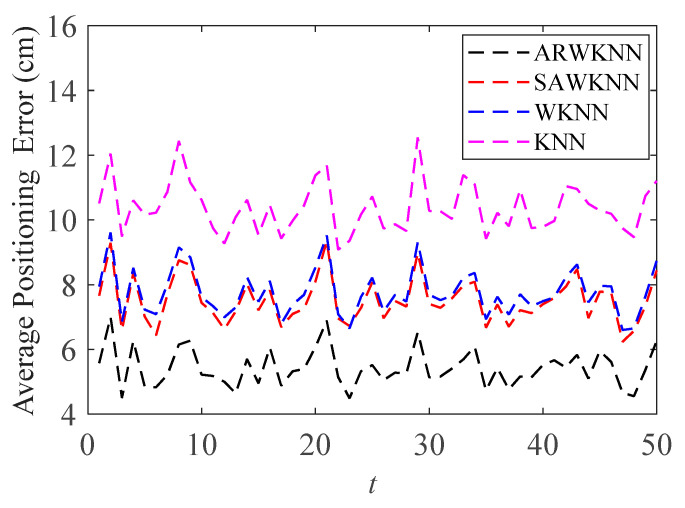
In 2-D, the impact of *t* on the average positioning error with *K*_max_ = 4.

**Figure 19:**
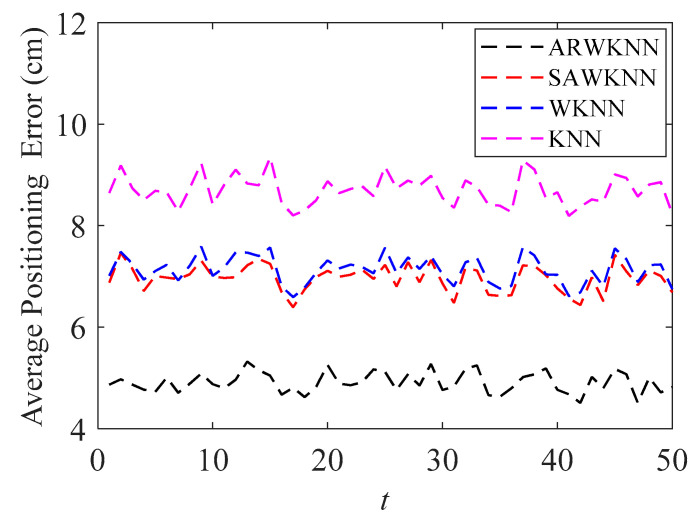
In 3-D, the impact of *t* on the average positioning error with *K*_max_ = 8.

When SNR = 20 dB, in order to analyze the robustness of the algorithm, fingerprints adopt non-uniform distribution structure, i.e., the RSSI values in the fingerprint map are chosen randomly at different sampling ratios *SR*. The average positioning errors of the ARWKNN, SAWKNN, WKNN, and KNN algorithms are analyzed with the variation of the fingerprint sampling ratio *SR*, the results of 2-D and 3-D are shown in Figure 16 and Figure 17, respectively. It can be seen that as *SR* increases from 50% to 100%, whether in 2-D or 3-D, the average positioning error based on the ARWKNN algorithm is significantly lower than that of the KNN, WKNN, and SAWKNN algorithms, and the larger the *SR*, the smaller the average positioning errors of the four algorithms. When *SR* = 50%, the average positioning errors of the ARWKNN, SAWKNN, WKNN, and KNN algorithms are analyzed with the variation of the time, and the results of 2-D and 3-D are shown in Figure 18 and Figure 19, respectively. It can be seen that as *t* increases from 1 to 50, whether in 2-D or 3-D, the average positioning error based on the ARWKNN algorithm is significantly lower than that of the KNN, WKNN, and SAWKNN algorithms. As can be seen from Figure 16, Figure 17, Figure 18 and Figure 19, the ARWKNN algorithm has good robustness. When the fingerprint sampling rate is only 50%, lower positioning errors can still be achieved.

The WKNN fingerprint positioning algorithm is based on the shortest RSSI physical distance between the fingerprint and the target position. It can be seen from Step 5 of the ARWKNN algorithm that the positioning error is affected by the weight of the fingerprint point and this weight is affected by the distance metric; therefore, it is necessary to analyze the impact of different distance metrics on the positioning error. In addition to Euclidean distance (ED) and Manhattan distance (MD), there are other distance metrics [12,37], such as:

Minimum maximum distance (MMD), which is defined as:

(16) $$dis_{l,m,n-k}=1-\frac{\sum_{i=1}^{M}\left(\min\left(\left|\phi_{l,m,n-i}\right|,\left|Y_{k,i}\right|\right)\right)}{\sum_{i=1}^{M}\left(\max\left(\left|\phi_{l,m,n-i}\right|,\left|Y_{k,i}\right|\right)\right)}$$

Squared Euclidean distance (SED), which is defined as:

(17) $$dis_{l,m,n-k}=\sum_{i=1}^{M}{\left(\phi_{l,m,n-i}-Y_{k,i}\right)}^{2}$$

Chebyshev distance (CHD), which is defined as:

(18) $$dis_{l,m,n-k}=\underset{i}{\max}\left|\phi_{l,m,n-i}-Y_{k,i}\right|$$

Squared-chord distance (SCD), which is defined as:

(19) $$dis_{l,m,n-k}=\sum_{i=1}^{M}{\left(\sqrt{\left|\phi_{l,m,n-i}\right|}-\sqrt{\left|Y_{k,i}\right|}\right)}^{2}$$

Wave hedges distance (WHD), which is defined as:

(20) $$dis_{l,m,n-k}=\sum_{i=1}^{M}\left(1-\frac{\min\left(\left|\phi_{l,m,n-i}\right|,\left|Y_{k,i}\right|\right)}{\max\left(\left|\phi_{l,m,n-i}\right|,\left|Y_{k,i}\right|\right)}\right)$$

Lorentzian distance (LD), which is defined as:

(21) $$dis_{l,m,n-k}=\sum_{i=1}^{M}\ln\left(1+\left|\phi_{l,m,n-i}-Y_{k,i}\right|\right)$$

Matusita distance (MTD), which is defined as:

(22) $$dis_{l,m,n-k}=\sqrt{\sum_{i=1}^{M}{\left(\sqrt{\left|\phi_{l,m,n-i}\right|}-\sqrt{\left|Y_{k,i}\right|}\right)}^{2}}$$

Squared chi-squared distance (SCSD), which is defined as:

(23) $$dis_{l,m,n-k}=\sum_{i=1}^{M}\frac{{\left(\phi_{l,m,n-i}-Y_{k,i}\right)}^{2}}{\left|\phi_{l,m,n-i}+Y_{k,i}\right|}$$

Canberra distance (CAD), which is defined as:

(24) $$dis_{l,m,n-k}=\sum_{i=1}^{M}\frac{\left|\phi_{l,m,n-i}-Y_{k,i}\right|}{\left|\phi_{l,m,n-i}+Y_{k,i}\right|}$$

Clark distance (CLD), which is defined as:

(25) $$dis_{l,m,n-k}=\sqrt{\sum_{i=1}^{M}{\left(\frac{\left|\phi_{l,m,n-i}-Y_{k,i}\right|}{\left|\phi_{l,m,n-i}+Y_{k,i}\right|}\right)}^{2}}$$

For different distance metrics, if the same *γ*_th_ value is used, the positioning error based on the SAWKNN algorithm will be greatly affected, so this section does not consider the SAWKNN algorithm. When SNR = 20 dB, we investigated 30 distance metrics and selected 12 distance metrics with the best performances, the results of which are shown in Table 4 and Table 5. It can be seen from Table 4 and Table 5 that when the KNN algorithm is used for positioning, ED and SED metrics produce the minimum average positioning error in 2-D and 3-D. In 2-D, the average positioning error based on the WKNN algorithm is similar to the experimental results in Alam et al. [12], we also get SCD and SCSD metrics produce the minimum average positioning error, but in 3-D, SED metric produces the minimum average positioning error. When the ARWKNN algorithm is used for positioning, the CLD metric produces the minimum average positioning error in 2-D and MMD metric produces the minimum average positioning error in 3-D. As far as the authors know, this is the first work to report the impact of CLD and MMD metrics on the positioning error of the fingerprint positioning algorithm. It can also be seen from Table 4 that the best values of the KNN, WKNN and ARWKNN algorithms are 4.84 cm, 2.03 cm and 1.45 cm, respectively. Compared with the KNN and WKNN algorithms, in 2-D, the minimum average positioning error of the ARWKNN algorithm can be reduced by 70.04%, and 28.57%, respectively. It can also be seen from Table 5 that the best values of the KNN, WKNN and ARWKNN algorithms are 4.46 cm, 3.05 cm and 2.18 cm, respectively. Compared with the KNN and WKNN algorithms, in 3-D, the minimum average positioning error of the ARWKNN algorithm can be reduced by 51.12%, and 28.52%, respectively. In 2-D or 3-D, the average positioning errors of the ARWKNN algorithm proposed in this paper are all smaller than that of the KNN and WKNN algorithms under 12 distance metrics.

**Figure 20:**
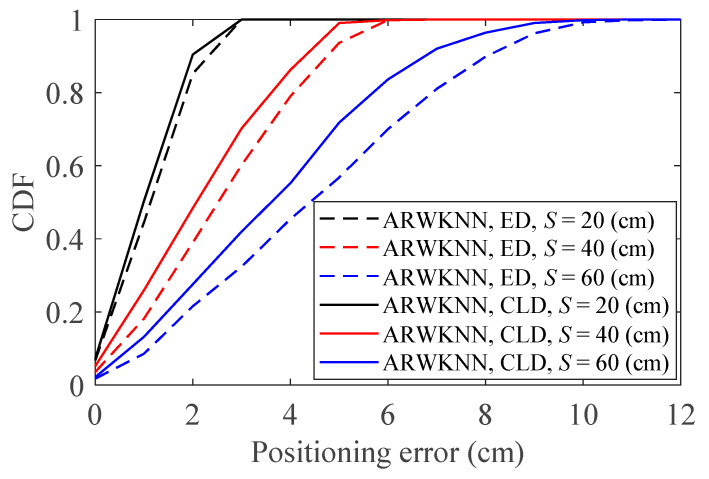
In 2-D, the cumulative distributions of positioning errors for the ED and CLD metrics with various *S* values. *K*_max_ = 4.

**Figure 21:**
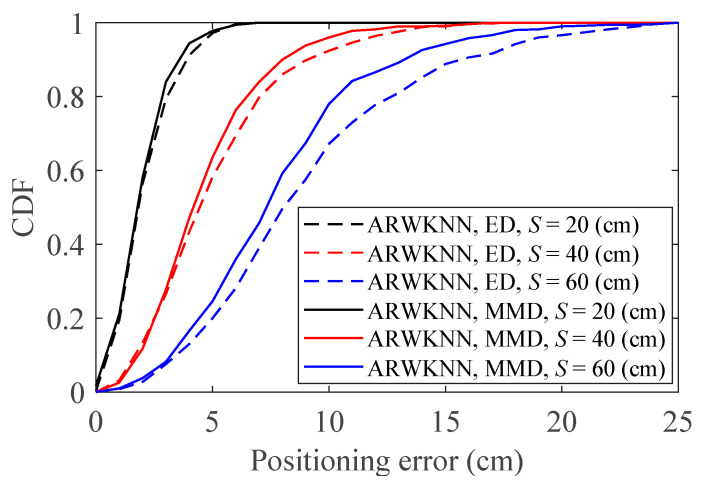
In 3-D, the cumulative distributions of positioning errors for the ED and CLD metrics with various *S* values. *K*_max_ = 8.

Figure 20 shows the cumulative distributions of positioning errors for the ED and CLD metrics with various *S* values. As can be seen from Figure 20, in 2-D, compared with the ED metric, the CLD metric produces smaller positioning error. In addition, compared with the CLD metric, the positioning error of the ED metric increases faster when *S* becomes larger. Figure 21 shows the cumulative distributions of positioning errors for the ED and MMD metrics with various *S* values. As can be seen from Figure 21, in 3-D, compared with the ED metric, the MMD metric produces smaller positioning error. In addition, compared with the MMD metric, the positioning error of ED metric increases faster when *S* becomes larger. ED is a commonly used distance metric, however, as can be seen from Table 4 and Table 5, in fact, the ED is not the most accurate metric for calculating weights when the WKNN and ARWKNN algorithms are used for positioning.

**Figure 22:**
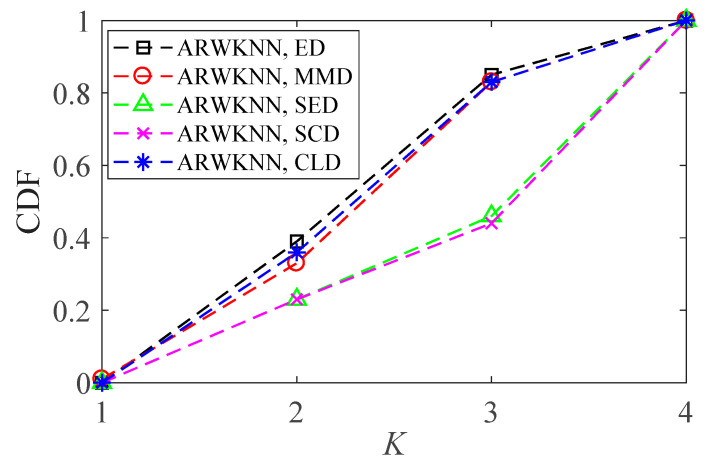
In 2-D, the cumulative distributions of the optimal *K* with *K*_max_ = 4.

**Figure 23:**
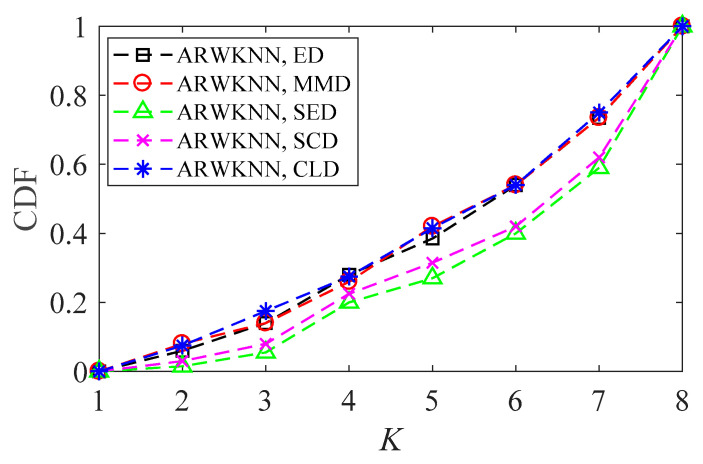
In 3-D, the cumulative distributions of the optimal *K* with *K*_max_ = 8.

SNR = 20 dB. To make the graph have a certain degree of discrimination, we only choose the ED, MMD, SED, SCD, and CLD metrics to analyze the cumulative distributions of optimal *K*. The results of 2-D and 3-D are shown in Figure 22 and Figure 23, respectively. As can be seen from Figure 22 and Figure 23, there are differences in the optimal *K* values for 200 targets, and there are also differences in cumulative distributions of the optimal *K* for five distance metrics. The optimal *K* cumulative distributions for ED, MMD and CLD are very close, and the optimal *K* cumulative distributions for SED and SCD are also very close.

The complexity of the KNN and WKNN algorithms mainly depends on the size of *N* and the sorting operation of Step 2 in Algorithm 1. Compared with the KNN and WKNN algorithms, the ARWKNN algorithm also performs Step 3 loop function and Step 4 min function in Algorithm 1. The time complexity of Step 3 plus Step 4 depends on the size of *K*_max_. Since *K*_max_ is much smaller than *N*, that is, the number of neighboring fingerprint points are much smaller than the total number of fingerprint points, the complexity of the ARWKNN algorithm is similar to the KNN and WKNN algorithms. In 3-D, when *K*_max_ = 8, the average computing time of 200 targets varying with *S* is analyzed, the result of which is shown in Table 6. It can be seen that when *S* is the same, the average calculation time of the KNN, WKNN, SAWKNN, and ARWKNN algorithms is almost the same. It can also be seen from Figure 13 that when *S* decreases, the average positioning errors of four algorithms decrease, but the complexity of the algorithm also increases. Therefore, according to the actual situation, the power consumption and positioning error of the algorithm can be compromised by selecting an appropriate *S*.

## 4. Conclusions

At present, the classical KNN and WKNN algorithms are mainly aimed at 2-D positioning, assuming that the height of the target from the floor is known, and it is not feasible to know the height of the target from the floor in advance. The least linear multiplication method and Newton–Raphson method are suitable for solving 2-D coordinates. Solving the 3-D coordinate is a non-convex optimization problem, which is easy to fall into a local optimal solution. In this paper, the shortcomings of the fingerprint positioning algorithm and the trilateration method are discussed, and an adaptive residual weighted *K*-nearest neighbor fingerprint positioning algorithm is proposed. Compared with the fingerprint positioning algorithm based on compressed sensing, the range-based WKNN algorithm can achieve high-precision positioning under the low-density LED layout. Compared with RF [14], ELM [16], ANN [17], and GI-LS [11] machine-learning algorithms, fingerprint positioning based on the ARWKNN algorithm not only has lower complexity, but also has lower positioning error. The impact of LEDs modulation bandwidth, LEDs transmit power, the signal-to-noise ratio, the maximum number of neighboring fingerprints, the sampling interval, the number of LEDs, the sampling ratio and distance metric on positioning errors are analyzed in detail. The distribution of optimal *K* and the complexity of the algorithm are also analyzed in detail. Simulation results show that the ARWKNN algorithm based on CLD and MMD metrics produces the smallest average positioning error in 2-D and 3-D, respectively. Compared with the SAWKNN [19], WKNN [12] and KNN [15,25] algorithms, the ARWKNN algorithm can significantly reduce the average positioning error while maintaining similar algorithm complexity.

Due to lighting requirements and LoS communication, the typical SNR of indoor visible light communication is relatively high, however, the RF, ELM, ANN, GI-LS, SAWKNN, WKNN, KNN, and ARWKNN algorithms have higher positioning error under low SNR conditions. Our next step is to design an efficient noise filtering algorithm to achieve higher positioning accuracy under low SNR conditions. LED communication can not only achieve high-precision positioning, but also achieve high rates. We will consider using modified OFDM to achieve high-precision positioning with high modulation bandwidth and provide a real scenario in the future.

## Figures and Tables

**Table 1:** The meaning of the indices.

Indices	Meaning
*m*	The number of columns corresponding to the fingerprint point
*n*	The number of rows corresponding to the fingerprint point
*l*	The number of dimensions corresponding to the fingerprint point

**Table 2:** Typical signal-to-noise ratio (SNR) in indoor environment.

	Minimum	Maximum	Average
SNR (*B* = 640 KHz)	42.97 dB	60.92 dB	52.45 dB
SNR (*B* = 100 MHz)	19.72 dB	37.35 dB	28.86 dB

**Table 3:** Average positioning error for each algorithm with SNR = 20 dB.

Algorithm	Average Positioning Error
ARWKNN	1.55 cm
RF	8.53 cm
ELM	9.65 cm
ANN	11.12 cm
GI-LS	3.89 cm
SAWKNN	2.76 cm
WKNN	2.97 cm
KNN	5.82 cm

**Table 4:** In 2-D, the impact of distance metrics on the average positioning error with *K*_max_ = 4, best values for KNN, WKNN, and ARWKNN algorithms are highlighted in bold.

Distance Metrics	KNN	WKNN	ARWKNN
ED	**4.84 cm**	2.61 cm	1.51 cm
MD	5.82 cm	2.97 cm	1.55 cm
MMD	5.84 cm	2.97 cm	1.54 cm
SED	**4.84 cm**	2.16 cm	1.85 cm
CHD	5.22 cm	2.97 cm	1.61 cm
SCD	4.99 cm	**2.03 cm**	1.82 cm
WHD	5.79 cm	2.94 cm	1.54 cm
LD	6.17 cm	3.45 cm	1.81 cm
MTD	4.99 cm	2.61 cm	1.50 cm
SCSD	4.99 cm	**2.03 cm**	1.82 cm
CAD	5.97 cm	2.93 cm	1.53 cm
CLD	5.05 cm	2.62 cm	**1.45 cm**

**Table 5:** In 3-D, the impact of distance metrics on the average positioning error with *K*_max_ = 8, best values for KNN, WKNN, and ARWKNN algorithms are highlighted in bold.

Distance Metrics	KNN	WKNN	ARWKNN
ED	**4.46 cm**	3.30 cm	2.31 cm
MD	4.63 cm	3.29 cm	2.28 cm
MMD	4.69 cm	3.33 cm	**2.18 cm**
SED	**4.46 cm**	**3.05 cm**	2.42 cm
CHD	5.18 cm	4.12 cm	2.86 cm
SCD	4.53 cm	3.13 cm	2.58 cm
WHD	4.91 cm	3.53 cm	2.43 cm
LD	5.30 cm	4.16 cm	2.61 cm
MTD	4.53 cm	3.41 cm	2.45 cm
SCSD	4.53 cm	3.13 cm	2.58 cm
CAD	4.88 cm	3.51 cm	2.41 cm
CLD	4.64 cm	3.58 cm	2.60 cm

**Table 6:** Computational complexity analysis.

Algorithm	The Value of *S*	Average Computing Time
KNN	*S* = 10 cm	15.07 ms
WKNN	*S* = 10 cm	15.18 ms
SAWKNN	*S* = 10 cm	15.51 ms
ARWKNN	*S* = 10 cm	15.28 ms
KNN	*S* = 20 cm	8.62 ms
WKNN	*S* = 20 cm	8.68 ms
SAWKNN	*S* = 20 cm	8.95 ms
ARWKNN	*S* = 20 cm	8.91 ms

## References

[1] Jang B., Kim H. (2019). Indoor positioning technologies without offline fingerprinting map: A survey. IEEE Commun. Surv. Tutor..

[2] Yao C.Y., Hsia W.C. (2018). An indoor positioning system based on the dual-channel passive RFID technology. IEEE Sens. J..

[3] Gharghan S.K., Nordin R., Jawad A.M., Jawad H.M., Ismail M. (2018). Adaptive neural fuzzy inference system for accurate localization of wireless sensor network in outdoor and indoor cycling applications. IEEE Access.

[4] Abdulrahman A., Abdulmalik A.S., Mansour A., Ahmad A., Suheer A.H., Mai A.A.A., Hend S.A.K. (2016). Ultra wideband indoor positioning technologies: Analysis and recent advances. Sensors.

[5] Zou H., Jin M., Jiang H., Xie L., Spanos C.J. (2017). WinIPS: WiFi-based non-intrusive indoor positioning system with online radio map construction and adaptation. IEEE Trans. Wirel. Commun..

[6] Yadav R.K., Bhattarai B., Gang H.S., Pyun J.Y. (2019). Trusted K nearest bayesian estimation for indoor positioning system. IEEE Access.

[7] Trong-Hop D., Myungsik Y. (2016). An in-depth survey of visible light communication based positioning systems. Sensors.

[8] Yuan P., Zhang T., Yang N., Xu H., Zhang Q. (2019). Energy efficient network localisation using hybrid TOA/AOA measurements. IET Commun..

[9] Wang Y., Ho K.C. (2018). Unified near-field and far-field localization for AOA and hybrid AOA-TDOA positionings. IEEE Trans. Wirel. Commun..

[10] Khalajmehrabadi A., Gatsis N., Akopian D. (2017). Modern WLAN fingerprinting indoor positioning methods and deployment challenges. IEEE Commun. Surv. Tutor..

[11] Guo X., Shao S., Ansari N., Khreishah A. (2017). Indoor localization using visible light via fusion of multiple classifiers. IEEE Photon. J..

[12] Alam F., Chew M.T., Wenge T., Gupta G.S. (2019). An accurate visible light positioning system using regenerated fingerprint database based on calibrated propagation model. IEEE Trans. Instrum. Meas..

[13] Lovón-Melgarejo J., Castillo-Cara M., Huarcaya-Canal O., Orozco-Barbosa L., García-Varea I. (2019). Comparative study of supervised learning and metaheuristic algorithms for the development of bluetooth-based indoor localization mechanisms. IEEE Access.

[14] Breiman L. (2001). Random forests. Mach. Learn..

[15] Xue W., Yu K., Hua X., Li Q., Qiu W., Zhou B. (2018). APs’ virtual positions-based reference point clustering and physical distance-based weighting for indoor Wi-Fi positioning. IEEE Internet Things J..

[16] Huang G.B., Zhou H., Ding X., Zhang R. (2012). Extreme learning machine for regression and multiclass classification. IEEE Trans. Syst. Man Cybern. B..

[17] Zhang S., Du P., Chen C., Zhong W.D., Alphones A. (2019). Robust 3D indoor VLP system based on ANN using hybrid RSS/PDOA. IEEE Access.

[18] Fang X., Jiang Z., Nan L., Chen L. (2018). Optimal weighted *K*-nearest neighbour algorithm for wireless sensor network fingerprint localisation in noisy environment. IET Commun..

[19] Hu J., Liu D., Yan Z., Liu H. (2019). Experimental analysis on weight *K*-nearest neighbor indoor fingerprint positioning. IEEE Internet Things J..

[20] Gu W., Aminikashani M., Deng P., Kavehrad M. (2016). Impact of multipath reflections on the performance of indoor visible light positioning systems. IEEE/OSA J. Lightwave Technol..

[21] Şahin A., Eroğlu Y.S., Güvenç I., Pala N., Yüksel M. (2015). Hybrid 3D localization for visible light communication systems. IEEE/OSA J. Lightwave Technol..

[22] Mathias L.C., Melo L.F.D., Abrao T. (2018). 3-D localization with multiple LEDs lamps in OFDM-VLC system. IEEE Access.

[23] Zhou B., Lau V., Chen Q., Cao Y. (2018). Simultaneous positioning and orientating for visible light communications: Algorithm design and performance analysis. IEEE Trans. Veh. Technol..

[24] Wu Y., Liu X., Guan W., Chen B., Chen X., Xie C. (2018). High-speed 3D indoor localization system based on visible light communication using differential evolution algorithm. Opt. Commun..

[25] Van M.T., Tuan N.V., Son T.T., Le-Minh H., Burton A. (2017). Weighted *k*-nearest neighbour model for indoor VLC positioning. IET Commun..

[26] Gligorić K., Ajmani M., Vukobratović D., Sinanović S. (2018). Visible light communications-based indoor positioning via compressed sensing. IEEE Commun. Lett..

[27] Tropp J.A., Gilbert A.C. (2007). Signal recovery from random measurements via orthogonal matching pursuit. IEEE Trans. Inf. Theory.

[28] Zhang R., Zhong W.D., Qian K., Zhang S., Du P. (2018). A reversed visible light multitarget localization system via sparse matrix reconstruction. IEEE Internet Things J..

[29] Feng C., Au W.S.A., Valaee S., Tan Z. (2012). Received-signal-strength-based indoor positioning using compressive sensing. IEEE Trans. Mob. Comput..

[30] Keskin M.F., Sezer A.D., Gezici S. (2018). Localization via visible light systems. Proc. IEEE.

[31] Zhou Z., Kavehrad M., Deng P. (2012). Indoor positioning algorithm using light-emitting diode visible light communications. Opt. Eng..

[32] Yasir M., Ho S.W., Vellambi B.N. (2014). Indoor positioning system using visible light and accelerometer. IEEE/OSA J. Lightwave Technol..

[33] Komine T., Nakagawa M. (2004). Fundamental analysis for visible-light communication system using LED lights. IEEE Trans. Consum. Electron..

[34] Zhang W., Chowdhury M.I.S., Kavehrad M. (2014). Asynchronous indoor positioning system based on visible light communications. Opt. Eng..

[35] Li H., Wang J., Zhang X., Wu R. (2018). Indoor visible light positioning combined with ellipse-based ACO-OFDM. IET Commun..

[36] Mossaad M.S.A., Hranilovic S., Lampe L. (2015). Visible light communications using OFDM and multiple LEDs. IEEE Trans. Commun..

[37] Cha S.H. (2007). Comprehensive survey on distance/similarity measures between probability density functions. Int. J. Math. Models Methods Appl. Sci..

